# Endoscopic combined esophageal semi-tunnel and gastric exposed resection for a large transesophagogastric leiomyoma: a case report

**DOI:** 10.3389/fmed.2026.1868258

**Published:** 2026-06-11

**Authors:** Lingfeng Ruan, Ling Zhang, Yiqian Liang, Tao Zhang

**Affiliations:** Department of Gastroenterology, Affiliated Hospital of Southwest Medical University; Nanchong Central Hospital, Luzhou, Sichuan, China

**Keywords:** case report, endoscopic resection, gastroesophageal junction, leiomyoma, subepithelial tumor

## Abstract

**Background:**

Resection of large subepithelial tumors at the gastroesophageal junction remains technically demanding because of the narrow operating space, the need to preserve junctional function, and the risk of postoperative reflux or stenosis.

**Case Presentation:**

A 36-year-old man was referred after a gastric cardia lesion was detected during routine endoscopy at an outside hospital. Contrast-enhanced CT with axial, coronal, and sagittal reconstructions showed a well-defined 30 × 18 mm lesion in the cardia-gastric fundus region. Endoscopy, however, revealed a subepithelial lesion extending from the distal esophagus across the gastroesophageal junction into the gastric cardia, suggesting a greater longitudinal extent than CT indicated. Endoscopic ultrasound had been performed at the outside hospital; however, the report did not clearly document the full longitudinal extent across the gastroesophageal junction. Because the lesion appeared low risk and the patient refused surgery, endoscopic resection was planned.

Under general anesthesia, endoscopy showed a subepithelial elevated lesion centered at the gastroesophageal junction, extending into the distal esophagus and gastric cardia. A gastric mucosal incision was made to expose the lesion, and an esophageal submucosal semi-tunnel was created from approximately 5 cm above the gastroesophageal junction and terminated above the cardia to preserve the mucosa. The two operative fields were then connected to achieve en bloc resection. The specimen measured 7 × 2 cm. Histopathology confirmed leiomyoma, and the postoperative course was uneventful.

**Conclusion:**

This case suggests that CT may underestimate the longitudinal extent of a transesophagogastric subepithelial lesion. In a selected case, a combined endoscopic esophageal semi-tunnel and gastric exposed resection approach with cardia mucosal preservation enabled en bloc resection. Further experience is needed to better define its indications and technical boundaries.

## Introduction

Subepithelial tumors of the upper gastrointestinal tract are frequently encountered during endoscopy. Although CT is useful for evaluating lesion location and adjacent organ relationships, it may underestimate the longitudinal extent of lesions with a predominantly intraluminal growth pattern (1). Endoscopic ultrasound (EUS) is the key tool for assessing the layer of origin and invasion depth of such tumors, and it may help differentiate leiomyoma from gastrointestinal stromal tumor; however, its performance is influenced by operator experience. For large lesions spanning the gastroesophageal junction, preoperative assessment and treatment planning remain challenging (2, 3).

For selected lesions, endoscopic submucosal dissection (ESD) and submucosal tunneling endoscopic resection (STER) are established minimally invasive treatment options. STER is generally used for muscularis propria tumors in the esophagus or cardia because it preserves the overlying mucosa. However, lesions that cross the gastroesophageal junction are more difficult to manage because the working space is limited and mucosal preservation at the cardia may be incomplete. Here, we report a large transesophagogastric leiomyoma removed using a combined endoscopic semi-tunnel and gastric exposed resection approach.

## Case presentation

A 36-year-old man was referred for a cardia lesion. Contrast-enhanced CT, including axial, coronal, and sagittal reconstructions, showed a well-defined 30 × 18 mm lesion in the cardia-gastric fundus region (Figure 1), but the exact craniocaudal length across the gastroesophageal junction could not be reliably measured. On retrospective review, this measurement mainly reflected the visible gastric component on CT, whereas the subtle extension across the gastroesophageal junction into the distal esophagus was not clearly delineated. Endoscopy revealed a subepithelial lesion centered at the gastroesophageal junction, extending from the distal esophagus into the gastric cardia, suggesting a greater longitudinal extent than initially appreciated on CT.

**FIGURE 1:**
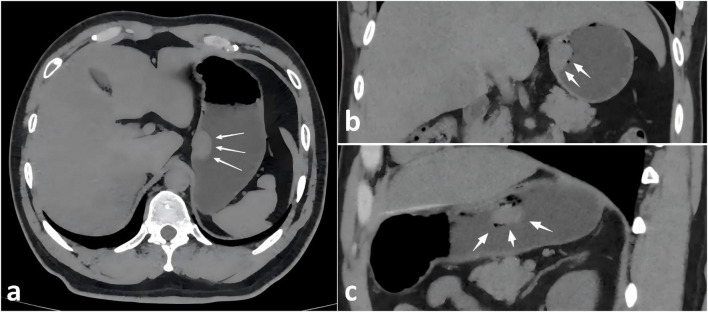
**(a)** Axial contrast-enhanced CT showing a low-density lesion in the cardia-gastric fundus region, measuring approximately 30 × 18 mm (arrows). **(b)** Coronal contrast-enhanced CT reconstruction showing the lesion in relation to the gastroesophageal junction (arrows). **(c)** Sagittal contrast-enhanced CT reconstruction showing the relationship of the lesion to the distal esophagus, gastroesophageal junction, and gastric cardia (arrows).

EUS had been performed at the outside hospital before admission. According to the outside-hospital medical record available to us, the lesion was described as a subepithelial lesion in the lower esophagus and subcardial region, with leiomyoma and gastrointestinal stromal tumor included in the differential diagnosis. However, the original EUS images were unavailable, and the documentation did not provide a detailed craniocaudal measurement across the gastroesophageal junction. Therefore, the full longitudinal extent could not be retrospectively verified. Because the lesion was considered low risk and the patient refused surgical resection, a minimally invasive endoscopic approach was selected, and a surgical backup plan was prepared.

The procedure was performed under general anesthesia with endotracheal intubation. A therapeutic gastroscope equipped with a transparent distal cap was used. After submucosal injection with saline mixed with indigo carmine and a small amount of epinephrine, a mucosal incision was created on the gastric side to expose the gastric component of the lesion. Submucosal dissection was then carried out along the tumor capsule. Endoscopy first showed a subepithelial elevated lesion centered at the gastroesophageal junction, extending to the cardia and distal esophagus (Figure 2a). Subsequently, an esophageal submucosal semi-tunnel was created from approximately 5 cm above the gastroesophageal junction and intentionally terminated above the cardia to preserve the cardia mucosa (Figure 2b). The esophageal and gastric operative fields were then connected under direct visualization, allowing en bloc resection of the lesion (Figure 2c and Supplementary Video 1). Minor bleeding was controlled endoscopically. The specimen was retrieved transorally after careful consideration of retrieval safety; no overtube or protective device was used. The mucosal defect was closed with endoscopic clips.

**FIGURE 2:**
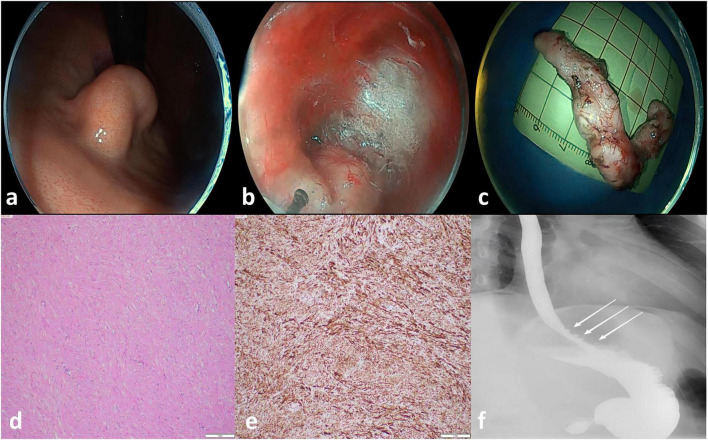
**(a)** Endoscopy revealing a subepithelial elevated lesion centered at the gastroesophageal junction, extending to the cardia and distal esophagus. **(b)** An esophageal submucosal semi-tunnel created from the esophageal side and terminated above the cardia, preserving the overlying mucosa. **(c)** Gross specimen of the completely resected tumor, measuring approximately 7 × 2 cm. **(d)** Hematoxylin and eosin staining showing spindle-shaped tumor cells ( × 20). **(e)** Immunohistochemical staining showing tumor cells positive for smooth muscle actin (SMA) and desmin, supporting the diagnosis of leiomyoma ( × 200). **(f)** Postoperative contrast radiography showing a patent esophagogastric tract without leakage (arrows).

Gross examination of the specimen showed a well-circumscribed spindle-shaped lesion measuring 7 × 2 cm (Figure 2c). Histopathology demonstrated spindle cells arranged in fascicles. Immunohistochemistry was positive for SMA, desmin, and H-caldesmon, and negative for CD117, DOG-1, and S-100. The Ki-67 index was < 1%, supporting a diagnosis of leiomyoma (Figures 2d, e). The pathology report did not separately describe margin status. Postoperative contrast radiography showed a patent esophagogastric tract without leakage (Figure 2f). The patient recovered uneventfully and was discharged on postoperative day 6 after routine observation and confirmation of esophagogastric passage patency. At 3-month follow-up, he remained asymptomatic.

## Discussion

This case suggests that CT may underestimate the longitudinal extent of a transesophagogastric lesion (1). In this patient, CT suggested a lesion of approximately 30 × 18 mm, whereas endoscopy demonstrated extension across the gastroesophageal junction. CT is useful for defining the gross location of a lesion and its relationship to adjacent structures, whereas endoscopy is better suited to assess mucosal involvement and the true luminal extent. When the findings are discrepant, EUS may help clarify the layer of origin and depth, although its accuracy depends on operator experience. In this case, EUS had already been performed at the outside hospital, and a subepithelial lesion had been recognized, but the available documentation did not clearly document the full longitudinal extent across the gastroesophageal junction.

The discrepancy between CT and endoscopic findings may be explained by several factors. First, the lesion showed a predominantly intramural/subepithelial growth pattern, which made its longitudinal boundaries difficult to distinguish on CT. Second, the lesion was located at the gastroesophageal junction, where the abrupt anatomical transition between the esophagus and cardia can obscure the true craniocaudal extent of a subepithelial tumor. In addition, the soft-tissue density of leiomyoma may be similar to that of the surrounding wall, further limiting CT in defining the full lesion length. For these reasons, CT may not reliably assess the longitudinal spread of transesophagogastric subepithelial lesions (1).

The rationale for choosing a combined endoscopic approach was based on the benign appearance of the lesion, the low-risk clinical impression, the patient’s refusal of surgical resection, and the desire to preserve the cardia mucosa. The term “semi-tunnel” refers to a limited submucosal tunnel created from the esophageal side that intentionally stops above the cardia to avoid mucosal damage at the gastroesophageal junction. “Gastric exposed resection” refers to gastric-side mucosal incision and direct endoscopic dissection of the lesion. A similar concept of preserving key muscular structures while reducing postoperative reflux has been reported in other submucosal endoscopic procedures (4).

STER is an established technique for selected muscularis propria tumors, particularly in the esophagus and cardia. Its main advantage is preservation of the overlying mucosa. However, it has limitations, including a restricted working space, reduced maneuverability for larger lesions, and difficulty when the tumor crosses the gastroesophageal junction. ESD can achieve en bloc resection in selected lesions, but it often creates a larger mucosal defect near the cardia. EFTR can provide complete resection, but the resulting full-thickness defect may be difficult to close securely at the gastroesophageal junction and may increase the risk of reflux or stenosis (1).

Compared with laparoscopic wedge resection or combined endoscopic-laparoscopic surgery, this approach avoids an external incision and may better preserve cardia function (2, 3). However, it is technically demanding and depends on lesion size, configuration, and operator experience. This combined technique may therefore be considered in selected cases when a lesion crosses the gastroesophageal junction and en bloc resection with mucosal preservation is desired. It should not be regarded as a universal standard.

## Conclusion

This case suggests that a transesophagogastric subepithelial lesion may extend beyond the dimensions estimated by CT alone. This is a single case report, and its findings should be interpreted cautiously. In selected larger lesions, a combined endoscopic esophageal semi-tunnel and gastric exposed resection approach may be considered for en bloc resection while preserving the cardia mucosa, but its use is limited by lesion size, configuration, and operator experience. Further experience is needed to better define its indications and technical boundaries.

## Data Availability

The original contributions presented in this study are included in this article/Supplementary material, further inquiries can be directed to the corresponding author.

## Supplementary material

Supplementary Video 1 Combined esophageal semi-tunnel and gastric exposed resection for a large transesophagogastric leiomyoma.
